# Biochemical and Functional Characterization of Anthocyanidin Reductase (ANR) from *Mangifera indica* L.

**DOI:** 10.3390/molecules23112876

**Published:** 2018-11-05

**Authors:** Lin Tan, Mei Wang, Youfa Kang, Farrukh Azeem, Zhaoxi Zhou, Decai Tuo, Lina María Preciado Rojo, Ikhlas A. Khan, Zhiqiang Pan

**Affiliations:** 1 Haikou Experimental Station, Chinese Academy of Tropical Agricultural Sciences (CATAS)—Hainan Key Laboratory of Banana Genetic Improvement, Haikou 570102, Hainan Province, China; farrukh@gcuf.edu.pk (F.A.); zhzx81@163.com (Z.Z.); 2 National Center for Natural Products Research, Thad Cochran Research Center, School of Pharmacy, University of Mississippi, University, MS 38677, USA; meiwang@olemiss.edu (M.W.); ikhan@olemiss.edu (I.A.K.); 3 Department of Basic Education in Liberal Arts, Hainan University, Haikou 571101, Hainan Province, China; kangyoufa@126.com; 4 Department of Bioinformatics and Biotechnology, Government College University, Faisalabad 38000, Pakistan; 5 Institute of Tropical Bioscience and Biotechnology, Chinese Academy of Tropical Agricultural Sciences (CATAS), Haikou 571101, Hainan Province, China; tuodecai123@163.com; 6 Programa Ofidismo-Escorpionismo, Facultad de Ciencias Farmacéuticas y Alimentarias, Universidad de Antioquia, Medellín 1226, Colombia; linampr@gmail.com; 7 United States Department of Agriculture, Agricultural Research Service, Natural Products Utilization Research Unit (USDA-ARS-NPURU), P.O. Box 1848, University, MS 38677-1848, USA; zhiqiang.pan@ars.usda.gov

**Keywords:** anthocyanidin reductase, (−)-epicatechin, (−)-catechin, proanthocyanidins, *Mangifera indica* L.

## Abstract

Mango (*Mangifera indica* L.) is abundant in proanthocyanidins (PAs) that are important for human health and plant response to abiotic stresses. However, the molecular mechanisms involved in PA biosynthesis still need to be elucidated. Anthocyanidin reductase (ANR) catalyzes a key step in PA biosynthesis. In this study, three ANR cDNAs (*MiANR1-1*,*1-2*,*1-3*) were isolated from mango, and expressed in *Escherichia coli*. In vitro enzyme assay showed MiANR proteins convert cyanidin to their corresponding flavan-3-ols, such as (−)-catechin and (−)-epicatechin. Despite high amino acid similarity, the recombinant ANR proteins exhibited differences in enzyme kinetics and cosubstrate preference. MiANR1-2 and MiANR1-3 have the same optimum pH of 4.0 in citrate buffer, while the optimum pH for MiANR1-1 is pH 3.0 in phosphate buffer. MiANR1-1 does not use either NADPH or NADH as co-substrate while MiANR1-2/1-3 use only NADPH as co-substrate. MiANR1-2 has the highest Km and Vmax for cyanidin, followed by MiANR1-3 and MiANR1-1. The overexpression of *MiANRs* in ban mutant reconstructed the biosynthetic pathway of PAs in the seed coat. These data demonstrate MiANRs can form the ANR pathway, leading to the formation of two types of isomeric flavan-3-ols and PAs in mango.

## 1. Introduction

Proanthocyanidins or PAs (also known as condensed tannin) belong to a subclass of plant metabolites called flavonoids. When incorporated into the diet, PAs can improve human health via their anticancer, anti-oxidant, cardiovascular protection and immunomodulatory effects [1,2,3,4,5,6,7]. These metabolites are widely distributed in stems, flowers, roots, tea-leaves, seed coats and fruits [8,9,10,11,12,13,14]. PAs play an important role in plant response to biotic/abiotic stresses like herbivore damage, fungal infection, mechanical wounding and ultraviolet irradiation [15,16]. Moreover, PAs can also affect plant traits of commercial interest, including skin color [17,18]. Therefore, it seems plausible to design strategies for increasing the levels of PAs in the fruits and vegetables of daily use.

**Figure 1:**
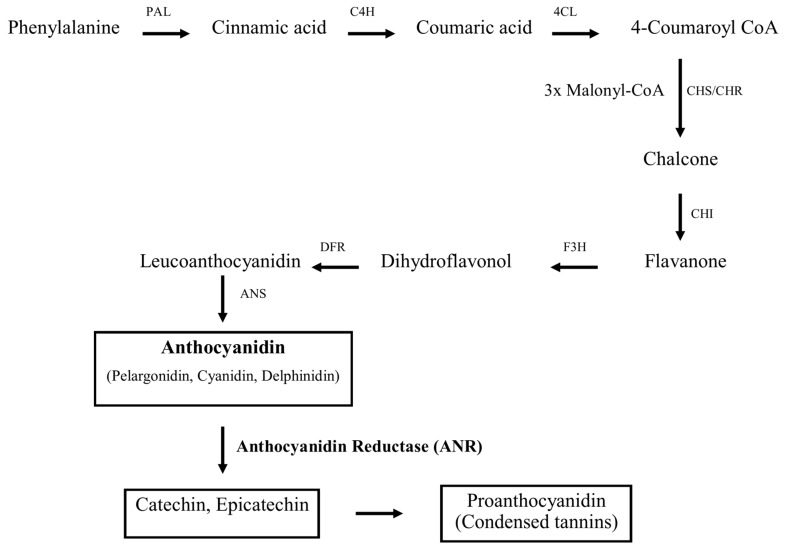
Scheme of the proanthocyanidin synthesis pathway.

The biosynthetic pathways leading to the formation of the monomeric units of PAs or falvan-3-ols (catechin and epicatechin) have been well studied in model plant species like maize [19] and *Arabidopsis* [20]. Flavan-3-ol monomers are synthesized via two distinct branches of the general flavonoid pathway, which share the same upstream biosynthetic pathway to leucocyanidin. The downstream pathway to PAs involves leucoanthocyanidin reductase (LAR) converting leucocyanidin into (+)-flavan-3-ols such as (+)-catechin [21] and anthocyanidin reductase (ANR), encoded by the *BANYLUS* gene in *Arabidopsis* [22], converting cyanidin into (−)-(epi)-flavan-3-ols, such as (−)-epicatechin (Figure 1).

The genes encoding ANR and LAR enzymes have been cloned and characterized in plants including poplar [23], buckwheat [10], lotus [24] and fruits such as grapevine [25], strawberry [26], persimmon [27] and apple [28]. The expression pattern of both genes is highly correlated with PA accumulation [26,27,28,29,30]. In apple, the transcript levels of *LAR1* and *ANR2* genes were significantly correlated with the contents of catechin and epicatechin [14]. In grape, during flowering, at the initial stage of berry growth and during colour-change in skins, the expression of *LAR* and *ANR* genes coincide with total tannin, catechin and epicatechin concentrations. It indicates potential involvement of ANR and LAR in PA synthesis [31].

Mango (*Mangifera indica* L.) is considered among the five most important fruit commodities traded worldwide, along with bananas, apples, grapes and oranges. It is very popular due to its excellent eating quality (bright color, sweet taste, luscious flavor), nutritional composition (vitamin, minerals) [32], fiber [33,34], and other phytochemical compounds including terpenoid [35], mangiferin [36] and flavonoids [37]. PAs (catechin and epicatechin) are one of important flavnoids in mango [34], and are major contributors to the total antioxidant capacity in mango fruit [38], which confer beneficial effect on human health. Besides, the antioxidant and antifungal properties of mango peel were associated with high levels of proanthocyanidins [39]. Therefore, it is very important to investigate the PA biosynthesis pathway in mango to improve its fruit quality.

The molecular mechanism underlying anthocyanin accumulation has recently been extensively studied in many fruits [40,41,42,43]. There are some studies which also reported the cloning of UFGT [44], F3′H [45] and DFR [46] genes related to anthocyanin synthesis from mango and their expression in red and green mango [47]. A mango fruit peel transcriptome in response to hot water treatment, also revealed anthocyanin biosynthesis-related genes [48]. However, there is little information available for genetic basis of PA biosynthesis pathway genes in mango.

In this report, we isolated three homolog cDNA sequences encoding ANR from mango. After the recombinant expression of these cDNAs in *E. coli* BL21, enzymatic activity was assayed in vitro. Moreover, enzymatic kinetics were also analyzed and compared. By over-expression in *Arabidopsis ban* mutant (ANR deficient) plants, the three ANR cDNAs were studied for potential complementation of ANR pathway leading to PA synthesis. This is the first report about the biochemical and transgenic characterization of ANR in mango, which lays a foundation for further investigations of the PA biosynthesis pathway in mango.

## 2. Results

### 2.1. Molecular Cloning of ANR Homolog from Hongguifei Mango

In the current study, 16 clones were obtained and sequenced, and after alignment analysis, three clones (that differed slightly in DNA sequence and amino acid sequence) were identified and named as MiANR1-1 (1289 bp), MiANR1-2 (1286 bp) and MiANR1-3 (1411 bp). Moreover, sequences were submitted to NCBI GenBank under the following accession numbers MG322128, MG322129, and MG322130, respectively. The ORFs encode 335 amino acids with a molecular weight (MW) of 36195 Da (pI: 5.91), 36339 Da (pI:5.46), 36304 Da (pI: 5.69), respectively.

**Figure 2:**
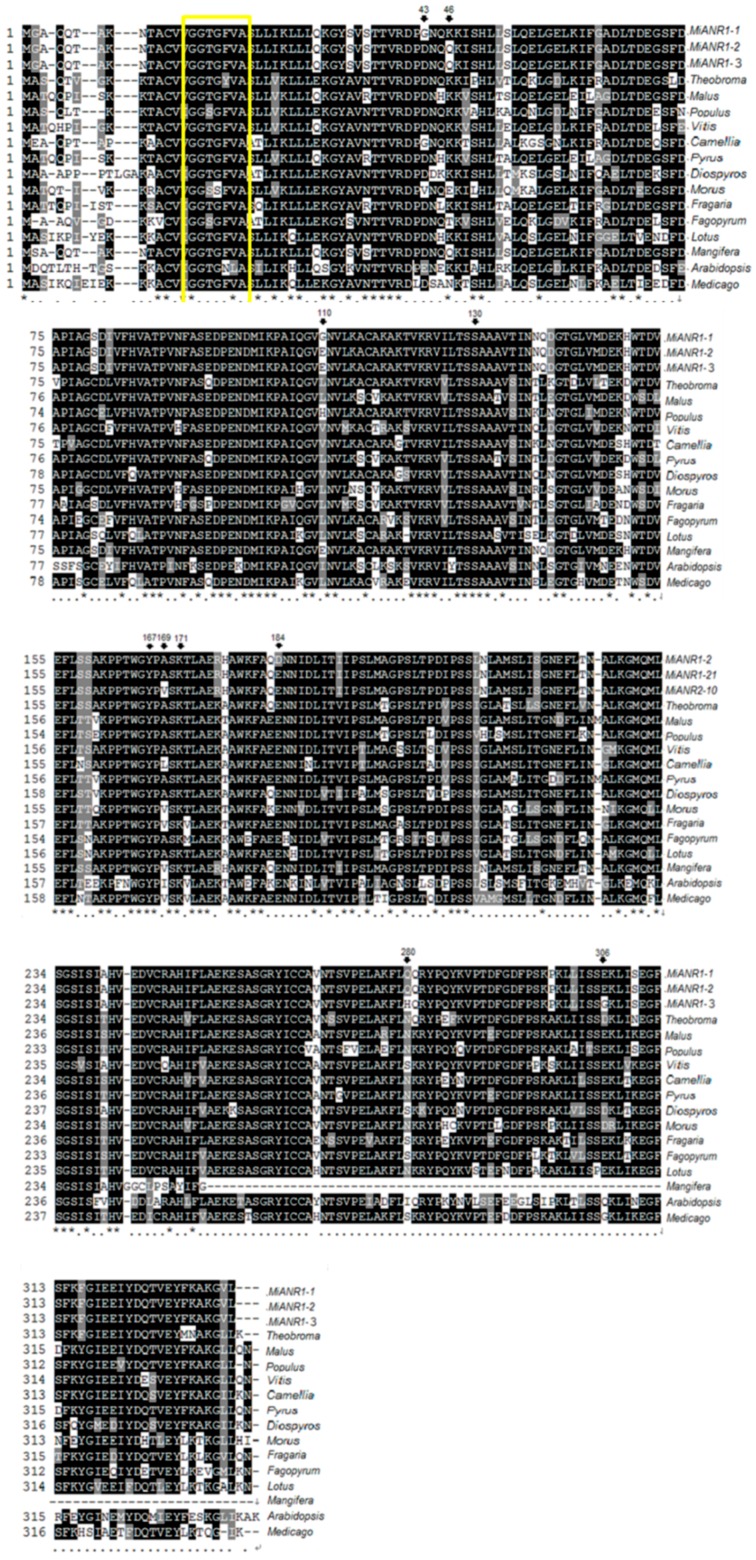
A sequence alignment of MiANR1-1,1-2 and 1-3 with ANR homologs from plants generated using Constraint-based Multiple Alignment Tool. *Mangifera* represents mango Iwin, * indicates the same amino acid in all 17 sequences. **.** shows semi-conservative amino acid in the 17 sequences. 43, 46, 110, 169, 184, 280, 306 indicates amino acid difference among MiANR1-1, 1-2 and 1-3.

**Figure 3:**
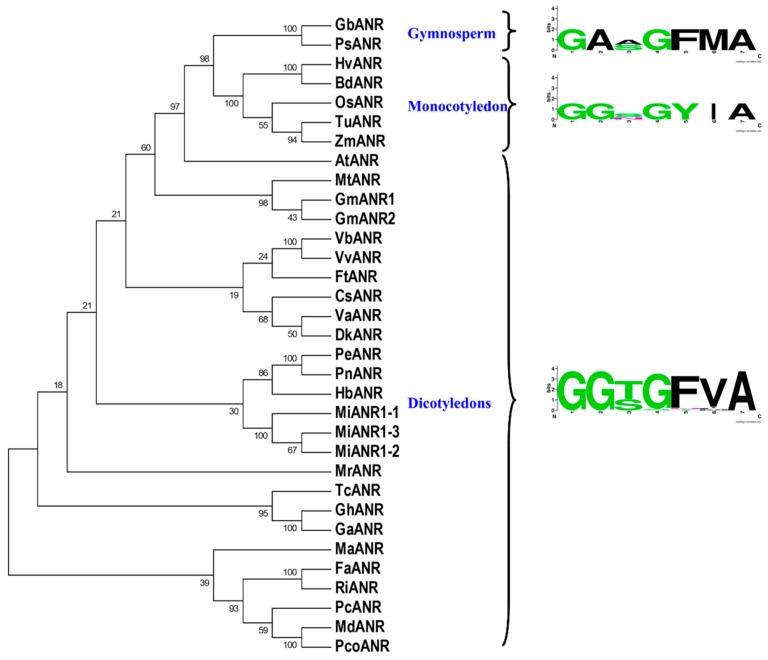
An unrooted phylogenetic tree with amino acid sequences of ANR homologs. Protein Alignment was performed by using ClustalW. The phylogenetic tree was constructed using neighbor-joining method with 1000 bootstrap iterations in Mega VII. **GbANR** (Q5XLY0.1, *Ginkgo biloba*); **PsANR** (ABR18365.1, *Picea sitchensis*); **ZmANR** (PWZ46070.1, *Zea mays*); **OsANR** (XP_015637099.1, *Oryza sativa*); **HvANR** (BAJ96327.1, *Hordeum vulgare*); **TuANR** (EMS66907.1, *Triticum Urartu*); **BdANR** (XP_003580614.1, *Brachypodium distachyon*); **VbANR** (AFG28175.1, *Vitis bellula*); **PeANR** (XP_011003389.1, *Populus euphratica*); **GhANR** (ABM64802.1, *Gossypium hirsutum*); **PcANR** (AKV89239.1, *Prunus cerasifera*); **GaANR** (NP_001316937.1, *Gossypium arboretum*); **TcANR** (ADD51354.1, *Theobroma cacao*); **MdANR** (AEL79860.1, *Malus domestica*); **HbANR** (XP_021671256.1, *Hevea brasiliensis*); **PnANR** (ART94427.1, *Populus nigra*); **VvANR** (NP_001267885.1, *Vitis vinifera*); **CsANR** (ADF43751.1, *Camellia sinensis*); **PcoANR** (AGL81352.1, *Pyrus communis*); **MrANR** (AIX02996.1, *Morella rubra*); **VaANR** (BAM42668.1, *Vaccinium ashei*); **DkANR** (BAF56654.1, *Diospyros kaki*); **MaANR** (ANR02605.1, *Morus alba var. multicaulis*); **FaANR** (ABG76842.1, *Fragaria x ananassa*); **FtANR** (AHA14497.1, *Fagopyrum tataricum*); **RiANR** (AMP19723.1, *Rubus idaeus*); **MiANR** (*Mangifera indica*); **AtANR** (Q9SEV0.2, *Arabidopsis thaliana*); **GmANR1** (NP_001241913.2, *Glycine max*); **GmANR2** (NP_001243072.1, *Glycine max*); **MtANR** (XP_013457149.1, *Medicago truncatula*).

An amino acid sequence alignment of 17 ANR homologs from 15 various plants was done by using Constraint-based Multiple Alignment Tool (COBALT, https://www.ncbi.nlm.nih.gov/tools/cobalt/cobalt.cgi? LINK_LOC=BlastHomeLink) (Figure 2). Results showed 98.81% amino acid sequence identity between MiANR1-1/1-2, 99.10% between MiANR1-2/1-3 and 97.91% between MiANR1-1 and 1-3. These three sequences exhibit differences at seven different amino acid residues. At three positions MiANR1-1/1-2 have same AA, while at four positions MiANR1-2/1-3 have identical AAs (Figure 2). Except for the AtANR sequence from *A. thaliana*, all the other ANR homologs shared highly conserved properties. Ser130, Tyr167 and Lys171 constitutes the putative enzymatic activity triad in all homologs [25,49]. Besides, a high conservation was also found at the glycine-rich Rossmann dinucleotide (NADPH/NADH)-binding domain (G-G-X-G-X-X-A) in these 17 homologs (Figure 2). In this domain sequence, GGTGFVA is conserved in all the ANRs from fruits, while it is variable among gymnosperms, monocotyledons and dicotyledon (Figure 3).

A phylogenetic analysis was conducted by amino acid sequences of ANR homologs from gymnosperms, monocotyledons and dicotyledons (Figure 3). The phylogenetic analysis showed that plant ANRs could be grouped into two main clades. One clade included the dicotyledon group, and the other included the gymnosperm group and the monocotyledon group, which suggested that monocotyledon ANRs were evolutionarily closer to gymnosperm ANRs than to dicotyledon ANRs. Among the three MiANRS, MiANR1-2 and MiANR1-3 fell into the same clade, indicating MiANR1-2 is evolutionarily closer to MiANR1-3 than MiANR1-1.

### 2.2. Functional Expression in E. coli and In Vitro Biochemical Characterization

**Figure 4:**
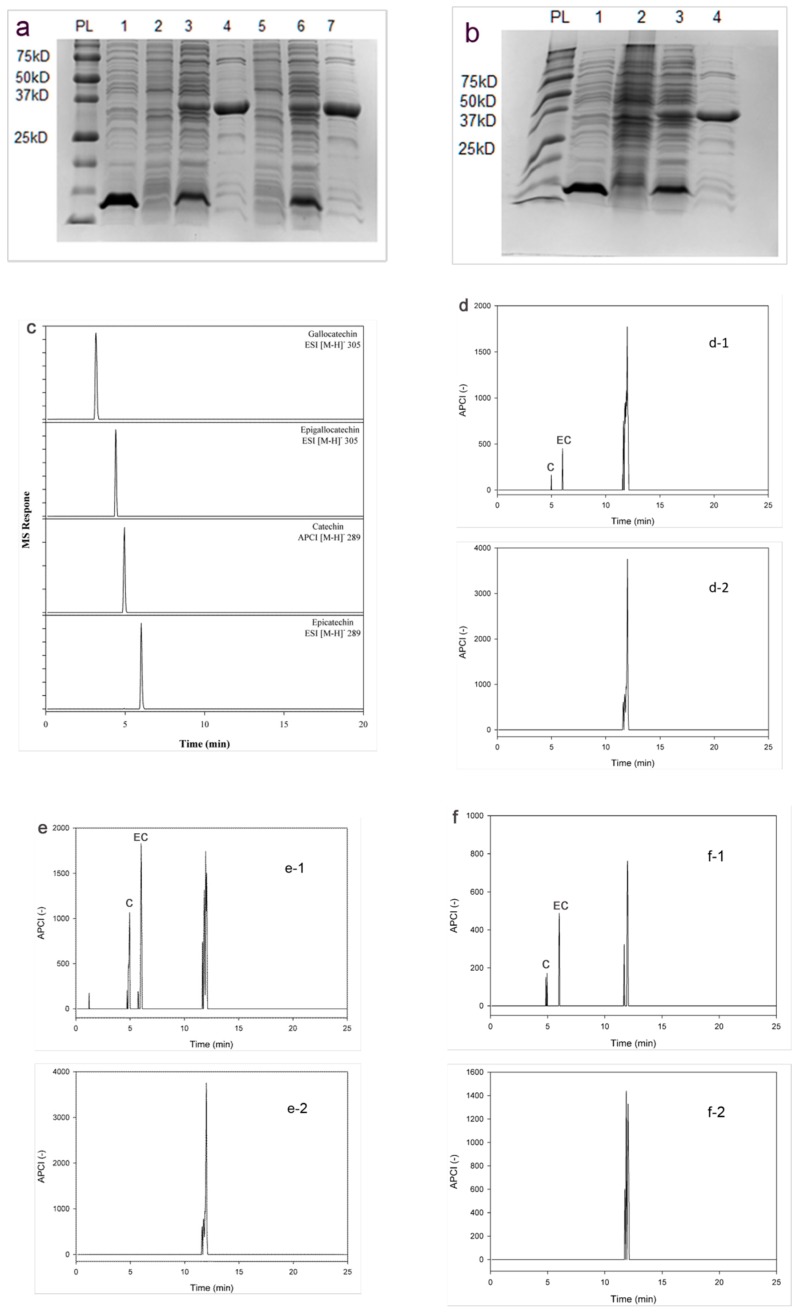

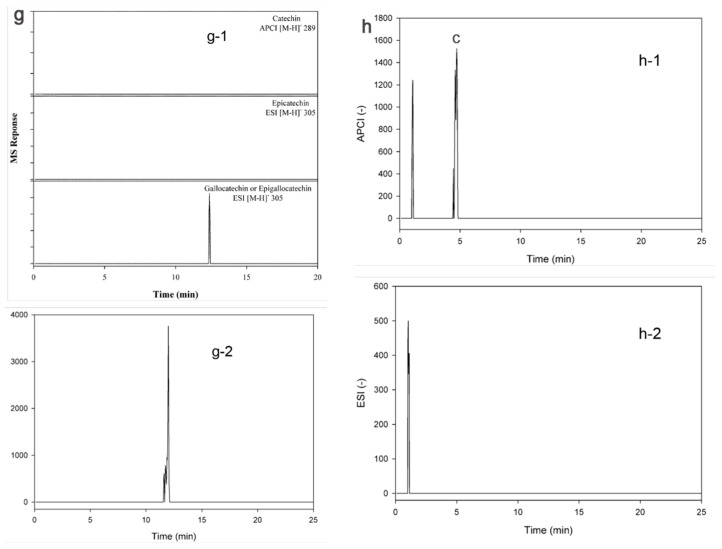
Recombinant expression of three MiANRs and their catalytic activity analysis. (**a**) SDS–PAGE (12%) gel electrophoresis of recombinant MiANR1-1 and MiANR1-2 proteins stained with Commassie brilliant blue G250. lane1: prestained protein mass markers (New England Biolabs), lane 2: 10 μg crude extract from *E. coli* BL21 harboring pET28b, lane 3:10 μg crude extract from uninduced BL21 harboring pET28bMiANR1-1, lane 4:10 μg crude extract from induced *E. coli* BL21 harboring pET28bMiANR1-1, lane 5:10 μg purified MiANR1-1, lane 6:10 μg crude extract from uninduced *E. coli* BL21 harboring pET28bMiANR1-2, lane 7:10 μg crude extract from induced *E. coli* BL21 harboring pET28bMiANR1-2, lane 7: 20 μg purified MiANR1-2; (**b**) lane 1: prestained protein mass markers(New England Biolabs); lane 2: 10 μg crude extract from *E. coli* BL21 harboring pET28b, lane 3:10 μg crude extract from uninduced BL21 harboring pET28bMiANR1-3, lane 4: 10 μg crude extract from induced *E. coli* BL21 harboring pET28b MiANR1-3, lane 5:10 μg purified MiANR1-3; (**c**) UPLC-MS profiles show authentic standard of catechin, epcatechin, gallocatecin, epigallocatechin; (**d**) UPLC-MS profiles show epicatechin and catechin formed from the incubation of MiANR1-1 and cyaniding (d-1) but not from that of denatured MiANR1-1 (control) and cyanidin (d-2); (**e**) UPLC-MS profiles show epicatechin and catechin formed from the incubation of MiANR1-2 and cyanidin (e-1) but not from that of denatured MiANR1-2 (control) and cyanidin (e-2); (**f**) UPLC-MS profiles show epicatechin and catechin formed from the incubation of MiANR1-3 and cyaniding (f-1) but not from that of denatured MiANR1-3 (control) and cyanidin (f-2); (**g**) UPLC-MS profiles shows nothing formed from the incubation of MiANR1-1 with delphinidin (g-1) and boiled MiANR1-1 with delphinidin (g-2). Data is not shown for MiANR1-2 and MiANR1-3; h UPLC-MS profiles show a peak formed from the incubation of MiANR1-2 or MiANR1-3 and perlargonidin (h-1) but not from that of denatured MiANR1-2 (control) with perlargonidin(h-2), however, it was not (−)-epi- afzelechin or (−)-afzelechin, as its MW is not the same as the MW of (−)-epi- afzelechin or (−)-afzelechin.

The ORFs of ANR cDNAs were cloned into pET28b vector and the recombinant *E. coli* Bl21 clones were treated with ITTG (50 µM) at 30 °C, resulting in high production of soluble recombinant protein. With a 6-His tag fusion to the C-terminal, the recombinant proteins were purified on Ni-NTA resin column from the Lysate. The size of recombinant MiANR1-1, MiANR1-2 and MiANR1-3 were identified as approximately 37 kD by using SDS-PAGE (Figure 4a,b). This expression system allowed for the production of 10–15 mg ANR protein per liter of culture.

To determine the enzymatic catalysis of MiANRs, three substrates (cyanidin, delphinidin and pelargonidin) were studied in the presence of either NADPH or NADH. All the three MiANR enzymes converted cyanidin into catechin and epicatechin with NADPH as coenzyme (Figure 4c–f). However, MiANRs were unable to use delphinidin and perlargonidin as a substrate (Figure 4g,h). In a reaction with NADH as coenzyme, MiANR1-2 and MiANR1-3 converted cyanidin into catechin and epicatechin (Figure S1b), while ANR1-2 converted cyanidin only into epicatechin (Figure S1c). All of them did not employ delphinidin and perlargonidin as substrate. In the control experiments with boiled enzyme for the reaction, no flavan-3-ols were produced from these three substrates.

### 2.3. Effects of Temperature, pH, and NaCl on ANR Activities

Based on the UHPLC results, a standard curve and corresponding equation (Figure S2) were established to calculate the production of enzymatic products for optimizing of pH and temperature values and characterization of kinetics.

**Figure 5:**
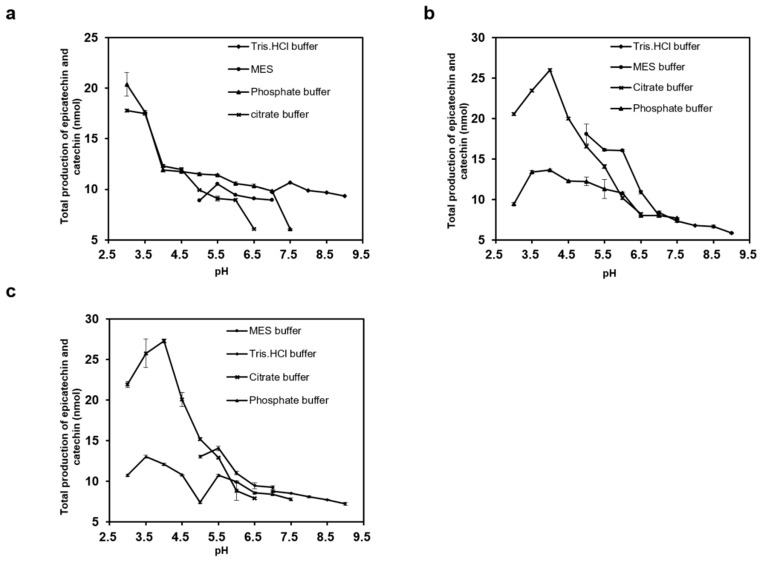
Effects of pH values on MiANR1-1, 1-2 and 1-3 activity. (**a**) pH effect on MiANR 1-1 activity tested by using Tris–HCl buffer, MES buffer, phosphate buffer and citrate buffer. (**b**) pH effect on MiANR 1-2 activity tested by using Tris–HCl buffer, MES buffer, phosphate buffer and citrate buffer. (**c**) pH effect on MiANR1-3 activity tested by using Tris–HCl buffer, MES buffer, phosphate buffer and citrate buffer. The total production includes all enzymatic products composed of epicatechin and catechin.

All these three enzymes exhibit their utmost efficiency in converting cyanidin to its corresponding flavan-3-ols at 30 °C (Figure S3). Variations were observed in pH dependence. All these three enzymes showed maximum activity at acidic pH. Both MiANR1-2 and 1-3 displayed the highest catalytic activity in citrate buffer at pH 4.0 but ANR1-1 gave the highest catalytic activity in phosphate buffer at pH 3.0 (Figure 5a–c). High sodium concentrations used in the buffers reportedly inhibited VbANR and MtANR [25,50]. NaCl (concentration ranging from 0–600 mM) did not cause any inhibition effect on the activity of MiANR1-1, 1-2 and 1-3, which is consistent with NaCl effect on the AtANR [51].

### 2.4. Kinetics of MiANRs with Coenzymes, Anthocyanidins and Perlargonidins

The production of enzymatic products exhibited a hyperbolic curve growth from 0 to 20 min but no significant increase at 30, 40 and 50 min. Hence, the 20 min was chosen as reaction time to investigate kinetics to coenzyme and substrates.

**Figure 6:**
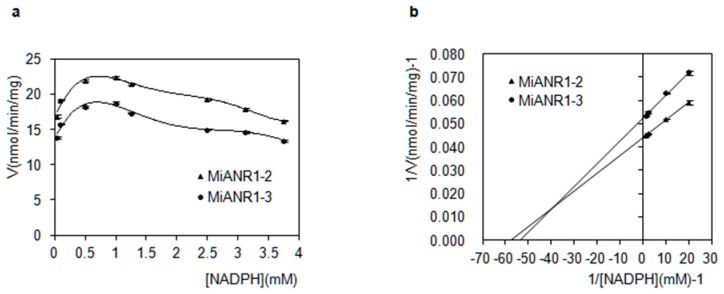
Kinetics of MiANR1-2, and 1-3 to NADPH. The initial velocity was expressed with nanomolar products produced from cyanidin per minute by per milligram of MiANR1-2/1-3 at varying NADPH concentrations. A plot of initial velocity versus [NADPH] for MiANR1-2/1-3 (**a**) and a double-reciprocal plot of 1/V versus 1/[NADPH]for MiANR1-2/1-3 (**b**).

Significant variations were found among MiANR1-1, 1-2 and 1-3 for co-substrate requirements. All the three enzymes did not use NADH as co-substrate. MiANR1-2 and 1-3 used NADPH as co-substrate, while MiANR1-1 did not use NADPH as co-substrate. Plots of the velocity (V) versus concentrations [NADPH] for MiANR1-2 and MiANR1-3 showed similar trend. At low concentration of NADPH (0–1 mM), their progress curves were almost linear (Figure 6a). From 1.25 to 3.75 mM, the velocity mildly decreased, indicating substrate inhibition. Double reciprocal plot of 1/V versus 1/NADPH for ANR1-2 and 1-3 were established by using initial velocity values to estimate Km and Vmax (Figure 6b). Results showed that MiANR1-2 has lower Km for NADPH (Table 1) than MiANR1-3 and higher Vmax than MiANR1-3, suggesting MiANR1-2 had a higher affinity to NADPH than MiANR1-3.

**Figure 7:**
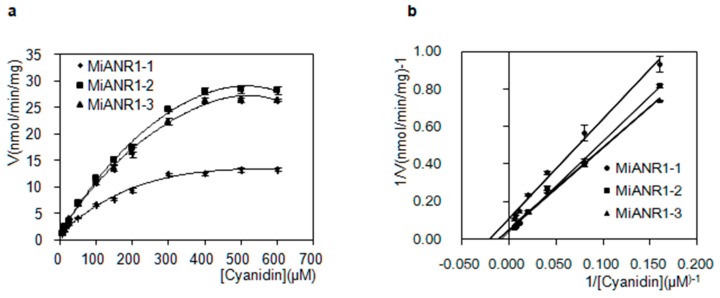
Kinetics of MiANR1-1, 1-2 and 1-3 with cyanidin. The initial velocity was expressed with nanomolar products produced from substrates per min by per milligram of ANR in the presence of NADPH (1 mM). Plots of initial velocity (V) versus cyanidin for MiANR1-1,1-2 and 1-3 (**a**) and double-reciprocal plots of 1/V versus 1/(cyanidin) for MiANRs (**b**). Products of cyanidin were calculated as (−)-epicatechin by using an (−)-epicatechin standard.

Variations were detected among properties of MiANR1-1, 1-2 and 1-3 towards anthocyanidin substrates. All the MiANRs showed much higher enzyme activity than VbANR [25], AtANR and MtANR [50]. They also exhibited classic Michaelis-Menten kinetics with cyanidin like VbANR and MtANR (Figure 7a). The double reciprocal plots indicated MiANR1-2 has the highest Vmax for cyanidin, followed by ANR1-3 and then ANR 1-1 (Figure 7b). This is consistent with UPLC-MS profiles showing the epicatechin and catechin formed from the incubation of MiANR1-2, MiANR1-3, MiANR1-1 and cyanidin.

### 2.5. Over-Expression of MiANR1-1, 1-2 and 1-3 in AtANR Mutant (Banyuls) Plants

**Figure 8:**
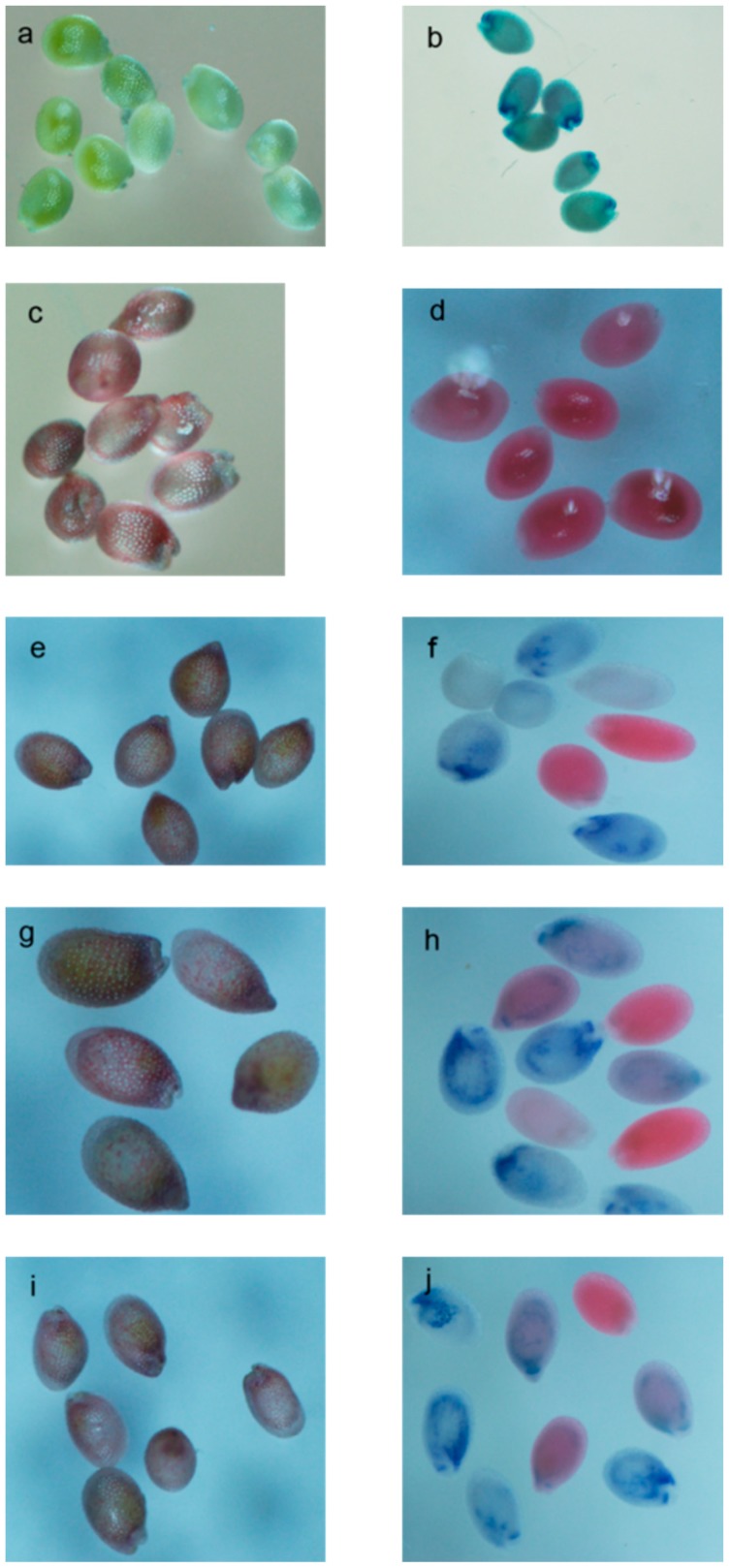
Genetic complementation of MiANR1-1, 1-2 and 1-3 expression in the *ban* mutant. Phenotypes of immature wild-type seeds (**a**), *ban* (**c**) and MiANR1-1, 1-2 and 1-3 transgenic ban (**e**,**g**,**i**) plants; staining with 0.1% DMACA to reveal the phenotypes of immature seeds from wild (**b**), *ban* (**d**), and MiANR1-1, 1-2 and 1-3 transgenic *ban* (**f**,**h**,**j**) plants, respectively.

*A. tumefaciens EHA*105 containing the pCB408 MiANR1-1, 1-2 or 1-3 was used to transform ANR mutant *Arabidopsis thaliana* plants (Figure S4). After transformation by the flower dip method, T0 seeds were obtained. To obtain T1 plants, plant selection was performed twice against the herbicide barstar resulting in herbicide resistant T1 plants which were further screened by PCR. PCR positive plants were transferred to pots to be cultivated. About six weeks later, the immature seeds were collected from transgenic, control *ban* and wild-type plants. Seeds were observed under microscope directly or after staining. Results showed that the immature seeds of wild type plants were lightly greenish. In contrast, the immature seeds from ban plants were reddish or pinkish due to the accumulation of anthocyanin in the endothelial layer of the seed coat (Figure 8c). It is because the mutated ANR [51,52] in the *ban* plants cannot convert anthocyanidin to proanthocyanidn. Surprisingly, the seeds from *MiANR 1-1,1-2* and *1-3* transgenic plant were also reddish or pinkish (Figure 8e,g,i). Further analysis by 0.1% DMACA staining indicated a bluish coloration of seed coats of wild type plants as DMACA can specifically react with proanthocyanidins. The stained seeds from *ban* plants showed deepened red color. Some of the stained seeds of *MiANR 1-1,1-2* and *1-3* transgenic *ban* showed bluish coloration, which suggested anthocyanidins converted into PAs by *MiANR 1-1,1-2* and *1-3* (Figure 8f,h,j). Due to gene segregation, some of the stained seeds of *MiANR 1-1,1-2* and *1-3* transgenic *ban* showed deepened reddish color like the *ban* seeds (Figure 8d) which is caused by the intensification effects of the 3 M HCl used in the DMACA solution. These results demonstrated that the over expression of MiANR1-1, 1-2 or 1-3 can reconstruct the ANR pathway to PAs.

## 3. Discussion

In this study, three cDNA clones encoding anthocyanidin reductase (MiANR1-1, MiANR1-2, and MiANR1-3) were isolated from *Mangifera indica*. All the clones were functionally expressed in *E. coli*, showing they encode active proteins and providing a way to investigate co-substrate/substrate specificity of the proteins in vitro. The protein sequence derived from the three clones contained the conserved Rossmann dinucleotide-binding domain amino-termini (G-G-X-G-X-X-A), which is responsible for binding nucleotide cofactors [53]. The amino acid of this domain of the three MiANRs is G-G-T-G-V-A, which is also shared by grape VbANR and legume MtANR. However, the three ANRs from mango exhibited different NADH/NADPH co-substrate specificity from VbANR and MtANR i.e., instead of using both NADH and NADPH as co-substrate, MiANRs use only NADPH as co-substrate. This is probably due to a mutation of Ser 2 to Ala in recombinant MiANRs caused by introduction of restriction enzyme site (CCATGG) to keep the integrity of open reading frame. Single amino acid change can alter cofactor binding specificity, for instance, mutation of Gln to Thr in *Rattus norvegicus* NADH dependent Cytochorome b5 reductase altered its cofactor specificity from NADH to NADPH [54]. Similarly, a mutation of Trp677 to Ala in rat NADPH-dependent cytochrome-P450 makes it into an enzyme with good NADH turn over [55]. However, it cannot be ruled out that other factors may also contribute to the NADH/NADPH preference like the hydrogen bond formation between the enzyme and 2′- phosphate of NADPH [56]. We used molecular docking to analyze the interaction between MiANR1-1, 1-2, 1-3 and NADH/NADPH (Figure S5). It was observed that there were more hydrogen bond formation between MiANR1-2 or MiANR1-3 and NADPH than their interaction with NADH. In addition, the binding free energy for the complex MiANR1-2 or MiANR1-3 with NADPH was higher compared to the complex with NADH. Besides, the conformation of NAD (P) H ligand may also affect the binding [57].

Unlike other ANRs (MtANR, AtANR, VbANR, CsANR, MdANR), which use cyanidin, perlargonidin and delphinidin as substrate, the MiANRs did not use delphinidin and perlargonidin as substrate. It indicates that MiANRs have unique substrate specificity. Similarly, dihydroflavonol 4-reductase (DFR) from different organisms exhibited different substrate specificity [58] and DFR homologs from the same organism (strawberry fruits) also revealed a high variability in substrate specificity [59]. This is the first report that an ANR has unique substrate specificity.

The three MiANRs also differed from those of individual ANRs from other plants in relative substrate preferences. For example, in *Medicago*, ANR shows preference for anthocyanidin substrates in the order of cyanidin > pelargonidin > delphinidin [58]. ANR in grape also exhibited the substrate preference in the order of cyanidin > pelargonidin > delphinidin [25], while ANR in *Arabidopsis* displays the reverse preference [58]. Compared to the above mentioned ANRs, both MiANR 1-2 and MiANR 1-3 showed substrate preference in the order of cyanidin > perlargonidin/delphinidin.

Similar to recombinant ANRs from legume [58], grape [25] and tea [11], all the three ANRs from mango convert cyanidin into (−)-epicatechin and (−)-catechin with similar Kcat/km. It suggests that cyanidin may be the major in vivo substrates, consistent with the red colored mango cultivars characterized with higher anthocyanins with cyanidin-3-O-monoglucosides as the major anthocyanins [60]. In contrast to other ANR proteins converting perlargonidin into (−)-epi-afzelechin and/or (−)-afzelechin, MiANRs does not use perlargonidin as substrate. It supports the fact that PA in mango is mainly composed of catechin and epicatechin [34,61].

In vitro enzyme assay showed that the three MiANRs shared similar biochemical properties. All of them have the same temperature and time length optima in the enzyme reaction and substrate/cosubstrate preference. While there are higher similarities between MiANR1-2 and MiANR1-3 than MiANR1-1 and MiANR1-3. For example, MiANR1-2 and 1-3 have the same optimum pH of 4.0 in citrate buffer. In contrast, the optimum pH for MiANR1-1 is 3.0 in phosphate buffer. MiANR1-2 and 1-3 also shared similar Km/kcat with cyanidin and have the same NADH/NADPH co-substrate preference. Based on the amino acid sequence alignment, despite these three MiANRs shared very high identity, MiANR1-2 exhibit higher amino acid sequence identity (99.1%) to MiANR1-3 than to MiANR1-1 (98.81%). That is probably why MiANR1-2 and MiANR1-3 revealed more similar biochemical properties than MiANR1-1 and 1-3.

Numerous investigations indicated that stereochemistry contributes a lot to the biochemical properties of flavan-3-ol. For example, (−)-catechin secreted from the roots of a destructive invasive weed *Centarea maculosa* Lam function as a broad-spectrum phyto-toxin. Unlike (−)-catechin, the (+)-catechin showed antibacterial activity against root-infesting pathogens. When consumed in diet, stereochemical configuration of epicatechin and catechin exhibited a profound influence on their uptake and metabolism in humans. Consequently, affect their beneficial effects on human health [62]. In this study, we did not use a chiral column to separate the enzymatic reaction products, but we can believe that the peaks from cyanidin and pelargonidin substrates were (–)-epicatechin, (−)-catechin, respectively. This is based on the investigations of Xie [22] who purified (–)-epicatechin, (−)-epiafzelechin, (−)-catechin, and (−)-afzelechin from a 500–1000 mL volume of ANR and substrate reactions at pH 7.0 by HPLC separation and identified their structure and configurations by NMR and CD spectrum analysis. Similar enzymatic assays were also conducted at the same pH 7.0 as described by Xie [22].

In this study, the seed coat from transgenic plants were pinkish like *banyuls* mutant seeds. It is due to the low level of production of proanthocyanidin in transgenic seeds, which turned bluish after staining with DMACA. It suggested that the expression of *MiANR1-1,1-2* and *1-3* in the seed is low. The formation of bluish color of the seed coat revealed the conversion of anthocyanidin into proanthocyanidin by MiANR1-1, 1-2 and 1-3 enzymes. It confirms that *MiANR1-1, 1-2* and *1-3* complemented the PA deficiency pathway. This is consistent with *VbANR* [25], *TcANR* [63] and GhANR [64] whose over-expression reconstructed the ANR pathway in *banuyls* mutant. Up to date, except mango ANR, only grape and apple fruit ANR has been transgenically characterized, although ANR cDNA homologs have been cloned or identified from a number of fruits like plum [64], pear [65], persimmion [27] and strawberry [25].

## 4. Materials and Methods

### 4.1. Plant Materials and Treatments

Hongguifei mango at the developmental stage (immature fruit) was collected from a mango farm located in Basuo Town, Dongfang County, Hainan Province, China. The pulp of Hongguifei mango was removed and quickly frozen in liquid nitrogen and then stored at −80 °C.

### 4.2. cDNA Cloning of ANR from Pulp of Hongguifei Mango by RACE

Fifty milligrams of frozen Hongguifei mango pulp at the developmental stage were ground into a fine powder in a mortar in liquid nitrogen. Total RNA samples free of genomic DNA were extracted from pulp powder using RNAprep Pure Plant Kit (Polysaccharide & Polyphenolics-rich) from Tiangen (Beijing, China). Degenerated primers (Table S1) of *ANR* gene were used for the amplification and cloning of a fragment of the mango *ANR* homolog. Then total RNA from Hongguifei mango pulp was used to synthesize the first strand cDNA as the protocol of the SMARTer^®^ RACE 5′/3′ Kit 5′and 3′cDNA suggested. The 5′end of ANR gene was amplified in two rounds of PCR with the gene-specific primers (Table S1) designed according to the above obtained fragments with the special sequence to the 5′end to facilitate infusion cloning. The 3′end of *ANR* gene was amplified in one round of PCR with the gene-specific primers (Table S1) designed according to the above obtained fragments with the special sequence to the 5′end to facilitate infusion cloning. After the 5′end and 3′end were sequenced, the synthesized 3′cDNA was used to clone the full length cDNA with the primers (Table S1) based on the sequenced 5′end and 3′end.

### 4.3. Expression of Recombinant MiANR cDNAs in E. coli

The selected the cDNA sequences were cloned into the MCS of pET28b without affecting the integrity of ORF. The forward primer (5′-CATGCCATG**G**CGGCCCAGCAAACGGCAA-3′) for cloning MiANR 1-1 included NcoI restriction site (underlined) with a mutation of T4 changed to G (bold face). The forward primer (CATGCCATG**G**CGGCCCA**A**CAAACGGCAA) for cloning ANR 1-2 and 1-3 also included NcoI (underlined) with a mutation of T4 changed to G (bold face). The three clones share the same reverse primer (5′-CCGCTCGAGCACTCCCTTAGCCTTGAAG-3′) with XhoI restriction site (underlined). Then PCR was carried out to amplify respective ORFs and the products were digested with restriction enzyme NcoI and XhoI, followed by purification with the DNA fragment Purification Kit (QIA quick ^®^PCR pufication kit). The purified cDNA were then ligated into the pET28b (Novagen, San Diego, CA, USA) vector that had already been digested by NcoI and XhoI. This ligation led to the fusion of cDNA to the C-terminal containing a His-tag-coding sequence in the vector, which is necessary for Ni-NIA affinity purification. Ligation products were introduced into TOP 10 competent bacteria. Positive colonies on an agar-solidified LB medium were selected to isolate and confirm the recombinant construct by double enzyme digestion and sequencing, and then named pET28bMiANR1-1, 1-2, 1-3 respectively.

To study protein expression, the recombinant vectors pET28bMiANR1-1, 1-2, 1-3, pET28bMiANR1-1, 1-2, 1-3 and an empty vector pET28b(+) (control) were introduced into ultra-competent cells of the Bl21 (De3) plysS (Invitrogen, Carlsbad, CA, USA) strain, respectively. A single positive colony was inoculated into 5 mL LB and grown overnight at 37 °C. The cultures were then diluted 1:100 and grown at 37 °C until the OD600 reached 0.6 (grown for 3 h). Then IPTG was added to a final concentration of 0.5 mm and further cultured at 30 °C and 220× *g* rpm overnight. The cells were harvested by centrifuge at 5000× *g* rpm at 4 °C for 15 min. Pellets were either used directly for enzyme extraction or stored at −80 °C.

### 4.4. Purification of Recombinant MiANRs

The purification was performed according to Qiagen Expressionist^TM^ (pp. 79, 81) with minor modifications. The harvested cells from 100 mL culture were suspended in 4 mL lysis buffer. Lysozyme was added as 2 mg/L, followed by incubation on ice for 30 min, sonication for 10 min and centrifugation at 10,000× *g* for 30 min at 4 °C to pellet the cellular debris. Then, 1200 μL of 50% Ni-NTA slurry was added to the clear lysate and mixed gently by shaking at 4 °C for 60 min. The lysate-Ni-NTA mixture was loaded into a column (10 mL) with the bottom outlet capped. The bottom cap was removed to collect the column flow-through, column was washed three times with 4 mL wash buffer, the protein was eluted 4 times with 0.5 mL elution buffer. At the end, the lysate, flow through, wash fraction, and eluted protein were collected for SDS-PAGE analysis.

### 4.5. Enzymatic Assay

Enzyme assays were initiated by mixing 140 μL Tris–HCl buffer (100 mM, pH7.0), 20 μL NADPH (final concentration: 1 mM), 20 μL of substrate (cyanidin, delphinidin, and perlargonidin, respectively, final concentration: 100 μM), 20 μL (20 ug) of purified recombinant MiANRs in a 200 μL reaction volume (with boiled purifed recombinant enzyme as control). The reactions were incubated at 37 °C for 45 min. All reactions were started by adding enzyme, and were stopped by adding 600 μL of ethyl acetate with 2 min of vigorous vortexing, and then centrifuged for 1 min at 12,000× *g* rpm. The ethyl acetate supernatant phase (500 μL) was transferred to a new Eppendorf tube, repeated the extraction step and pooled the two ethyl acetate extractions (1000 μL), which was dried by using a speedy vacuum at room temperature. Residues were dissolved in 60 μL HPLC grade methanol for UHPLC-UV-MS analysis.

### 4.6. Determination of Kinetic Parameters for Recombinant MiANRs

The following experimental steps were specified for obtaining an appropriate reaction time, the optimization of pH value and temperature, the kinetic characterization of substrates and NADPH/NADH, and NaCl effect on MiANR activity. All enzymatic reactions were stopped and extracted by adding ethyl acetate as described above. Extraction steps and sample preparation for UV spectrometry analysis were described below.

First, several different buffer systems including citrate buffer (pH 3.0–6.5 100 mM), phosphate buffer (pH 3.0–7.5 100 mM), Tris–HCl buffer (pH 7.0–9.0 100 mM) and 50 mM MES (5.0–7.0) buffer were tested to get the optimal pH value. Each single enzymatic reaction consisted of 70 μL of buffer, 10 μL of cyanidin chloride (final concentration: 100 μM), 10 μL of NADPH (final concentration: 1 mM), and 10 μL of MiANR protein (final amount: 10 μg). Control reactions were performed using BSA. All reactions were conducted at 40 °C for 45 min.

The temperature profiles for MiANRs were determined at their respective optimal pH value of phosphate buffer 3.0 (ANR1-1), citrate 4.0 (ANR1-2), citrate 4.0(ANR1-3) with 45 min incubations in a final volume of 100 μL containing 70 μL buffer, 10 μL of cyanidin chloride (final concentration: 100 μM), 10 μL of NADPH (final concentration: 1 mM), and 10 μL of MiANR protein (final amount: 10 μg). The tested reaction temperatures included 30, 34.6, 39.5, 45.3, 49.6 and 55 °C, which are performed by gradient PCR.

The optima reaction time was determined at the respective pH optima and temperature optima in total volume of 100 containing 70 μL buffer, 10 μL of cyanidin chloride (final concentration: 100 μM), 10 μL of NADPH (final concentration: 1 mM), and 10 μL of MiANR1-1, 1-2 and 1-3 protein (final amount: 10 μg). The tested times included 2, 5, 8, 10, 20, 30, 40, and 50 m.

The kinetics of recombinant MiANRs with NADPH were characterized at the respective pH, temperature and time optima in total volume of 100 μL containing 70 μL of buffer, 10 μL of cyanidin chloride (final concentration: 100 μM), 10 μL of NADPH (different concentrations), and 10 μL of ANR protein (final amount: 10 μg). The tested concentrations of NADPH include 0 (control), 0.05, 0.1, 0.5, 1, 1.25, 2.5, 3.125, and 3.75 mM. The kinetic properties of recombinant MiANRs were likewise determined in total volume of 100 μL containing 70 μL buffer (100 mM), 10 μL of cyanidin chloride (0, 6.25, 12.5, 25, 50, 100, 150, 200, 300, 400, 500 and 600 mM), and 10 μL NADPH (final concentration: 1 mM), ANR1-1, 1-2, 1-3 protein (final amount: 10 μg).

To determine the effects of NaCl concentrations, enzyme assays were carried out at the respective pH, temperature, time optima in total volume of 100 μL consisting of 60 μL of buffer, 10 μL of cyanidin chloride (final concentration: 100 μM), 10 μL of 1 mM NADPH, 10 μL of ANR protein and 10 μL of NaCl (0–500 mM). All the control reactions were performed using BSA.

### 4.7. UHPLC-UV-MS and Ultraviolet–Visible Spectrometry Analyses

An UHPLC-MS method was developed for the simultaneous analysis of the four compounds, viz. catechin [8], epigallocatechin [9], epicatechin [10], and gallocatechin [11]. The analyses were performed on an Agilent 1290 Infinity series LC (Agilent, Santa Clara, CA, USA) that included a binary solvent manager, sampler manager, thermostatted column compartment, diode array (DAD) detector. The LC instrument was coupled to an Agilent 6120 quadrupole mass spectrometer with a dual APCI and ESI interface. The column was an Agilent ZORBAX Eclipse Plus C_18_ (2.1 × 100 mm, 1.8 µm) column. The column temperature was maintained at 30 °C. The eluent consisted of water with 0.05% formic acid (A) and acetonitrile in 0.05% formic acid (B). Analysis was performed using the following gradient elution at a flow rate of 0.25 mL/min: 0–10 min, 5% B to 30% B; 10–15 min, 30% B to 60% B; and increasing B to 100% in next 3 min. The analysis was followed by a 7 min washing procedure with 100% B and re-equilibration period of 6 min. All solutions were filtered through 0.45 µm PTFE filters and the injection volume was 5 µL.

Both ESI and APCI sources were evaluated in the positive and negative modes to scan a mass range of 100–800. Mass spectrometer conditions were optimized to obtain maximal sensitivity. The fragmentor voltage was 100 V, and the capillary voltage was 4000 V. The drying gas flow rate was 10.0 L/min, the nebulizer pressure was 30 psi, and the drying gas temperature was 300 °C. Signals in negative modes at *m*/*z* 289 [M − H]^−^ (APCI), 289 [M − H]^−^ (ESI), 305 [M − H]^−^ (ESI) and 305 [M − H]^−^ (ESI) were used to detections of compounds gallocatechin, epigallocatechin, catechin and epicatechin, respectively.

An ultraviolet–visible (UV) spectrometry analysis was employed to quantify the enzymatic reaction products. This method is based on dimethylaminocinnamaldehyde (DMACA) reacts with flavan-3-ols producing deep bluish compounds, which have a maximum absorption at 640 nm [66,67,68]. First, the standard curves and equations were developed to calculate flavan-3-ols from enzymatic reactions as described by [25]. Then 30 μL of crude methanol extract from each enzymatic reaction was mixed with 30 μL of 0.1% DMACA in a 96 well plate, which was then kept in room temperature for 10 min, followed by absorbance measurement at 640 nm (BioTek Microplate Reader ELX808, Winooski, VT, USA). The blank with BSA in place of recombinant MiANR1-1, 1-2, 1-3 was likewise assayed. This UV spectrometry method was used for optimizing the pH and temperature values, analyzing the kinetics and the effects of sodium chloride. Each experiment was repeated at least three times. The plots of the initial reaction velocities versus the substrate concentrations were established by using the data from UV spectrometric analyses. All Km, Vmax, and Kcat values related to substrates and NADPH/NA DH were obtained by using values from the UV spectrometry measurement.

### 4.8. Over-Expression of MiANR in the Banuyls Mutant of Arabidopsis thaliana

A pair of primers (Forward primer: 5′-CCGGAATTCAAATCAACCATGTCGGCCCA-3′ including an EcoRI restriction site, Reverse primer: CCGCTCGAGTCATTAGAGCACTCCCTTA GCCTTG including an XhoI restriction site) were designed to amplify the ORFs of MiANR cDNA, which were recovered using the recovery kit and ligated to the double digested pub vector (constructed by Zhiqiang Pan) to produce pub MiANR. Then pubMiANR and plant expression vector pCB408 were digested with SfiI respectively. The fragments containing the ORF were recovered and cloned into pCB408 vector. The recombinant binary vectors, named pCB408MiANR1-1, 1-2, 1-3 were obtained and then introduced into *Agrobacterium tumefaciens* strain EHA105 by using a modified protocol of the freeze-thaw method [69]. The positive colonies were selected by colony PCR and then used to transform the *banuyls* (*ban*) mutant line.

Mutant plants (250C and 474C) were cultivated in a growth chamber with a 12/12 photoperiod at 22 °C. Leaves were collected to extract DNA to perform PCR to indentify the genetic purity of the mutant. When the floral stems were about 10 cm high, the floral dipping transformation was done according to the protocol described by Logemann [70]. Transformed plants were kept in the plant growth chamber with Columbia wild-type and ban mutants (not transformed) grown in the same condition as control. Seeds were obtained upon full maturation and germinated on soil. One week after germination, 0.1% BASTA herbicide was sprayed to screening the transgenic plants in the plant growth chamber and repeated twice at seven days intervals. Transgenic plants were identified if they continued to grow and stayed green. In contrast, the untransformed plants kept small, went white and died two weeks after selection. Simultaneously, PCR was also used to confirm the transgenic plants. Green siliques were collected from wild, transformed and non-transformed control plants. Immature seeds were removed from the siliques to be observed under microscope and photographed, and then stained with 0.1% DMACA as described by Xie [22]. After 20 min of staining, they were observed under dissecting microscope and photographed for their coloration.

## 5. Conclusions

We have cloned and functionally characterized three ANR cDNAs genes from Hongguifei mango in vitro and in vivo. All of them have the ability to reduce anthocyanidin into proanthocyanidins, while they differ from ANRs of other plants like *Arabidopsis* and *vitis vibro*, in co-substrate/substrate specificity and enzyme kinetics. This study has laid a foundation for further investigating the regulation of PAs biosynthesis in mango.

## Figures and Tables

**Table 1:** Kinetic properties of Mi ANR1-1, 1-2and 1-3.

Enzyme		Substrates	Cofactors
		Cyanidin	NADPH
MiANR1-1	Vmax (nmol/min mg)	9.11 ± 0.15	NR
	Km (μM)	48.32 ± 3.45	-
	Kcat (min^−1^)	958	-
	Kcat/Km (M^−1^ S^−1^)	3.3 × 10^5^	-
MiANR1-2	Vmax (nmol/min mg)	24.15 ± 0.92	22.68 ± 0.18
	Km (μM)	116.4 ± 4.42	18.14 ± 1.4
	Kcat (min^−1^)	2542	-
	Kcat/Km (M^−1^ S^−1^)	3.63 × 10^5^	-
MiANR1-3	Vmax (nmol/min mg)	18.42 ± 1.38	18.98 ± 0.1
	Km (μM)	80.43 ± 5.85	18.98 ± 1.18
	Kcat (min^−1^)	1938	-
	Kcat/Km (M^−1^ S^−1^)	4.01 × 10^5^	-

NR: No reaction.

## Supplementary Materials

The following are available online. Table S1. And Figures S1–S5.
