# Supplementary material for: Biochemical and Functional Characterization of Anthocyanidin Reductase (ANR) from Mangifera indica L
Source: Molecules. 2018 Nov 5;23(11):2876. doi: 10.3390/molecules23112876 (PMC6278290; doi:10.3390/molecules23112876)
Supplement: Supplementary file 1 [file molecules-23-02876-s001.pdf]

Supplementary material

cDNA Cloning of *ANR* from pulp of Hongguifei Mango by RACE

Degenerated primers (Table S1) were designed using the aligned sequences of anthocyanidin reductase genes (from other plants). It allowed the amplification and cloning of a fragment of the mango *ANR* homolog. Then total RNA from Hongguifei mango pulp was used to synthesize the first strand cDNA as suggested by the protocol of SMARTer® RACE 5′/3′ Kit 5′and 3′cDNA. The 5′end of *ANR* gene was amplified in two rounds of PCR with the gene-specific primers (Table S1) designed according to the above obtained fragments with the special sequence to the 5′end to facilitate infusion cloning. The 3′end of *ANR* gene was amplified in one round of PCR with the gene-specific primers (Table S1) designed according to the above obtained fragments with the special sequence to the 5′end to facilitate infusion cloning. After the 5′end and 3′end were sequenced, the synthesized 3′cDNA was used to clone the full length cDNA with the primers (Table S1) based on the sequenced 5′end and 3′end.

Table S1. List of primers for cDNA cloning of ANR from Hongguifei mango

Name	Oligonucleotide sequence 5′-3′	Application
DG-ANR1	GGACCCGGAGAATGATATGAT	PCR for mid
DG-ANR2	CTTTGCCTTGAAGTACTCCAC	PCR for mid
ANR405R-In	GATTACGCCAAGCTTGGTCACAGCAGCAGCTGAAGATGTCAATATGACTC	The first 5 RACE
ANR350R-In	GATTACGCCAAGCTTGCACAGGCTTTCAAAACATTCTCTACTCCTTGGAT	The second 5 RACE
ANR387F-In：	GATTACGCCAAGCTTTTCAGCTGCTGCTGTGACCATCAATAACCAAGATG	3′RACE
ANR5-F1	AGTCCCATCGAATCTGTGTCATTCTTAGC	Full cDNA
ANR5-F2	AAGCCAAGGTTGAGTCGTTGGTGGTCCT	Full cDNA
ANR3-R	GTAGCAAATTTATATTTATTTCATTCACATTAGC	Full cDNA

MiANRs catalytic activity with three substrates

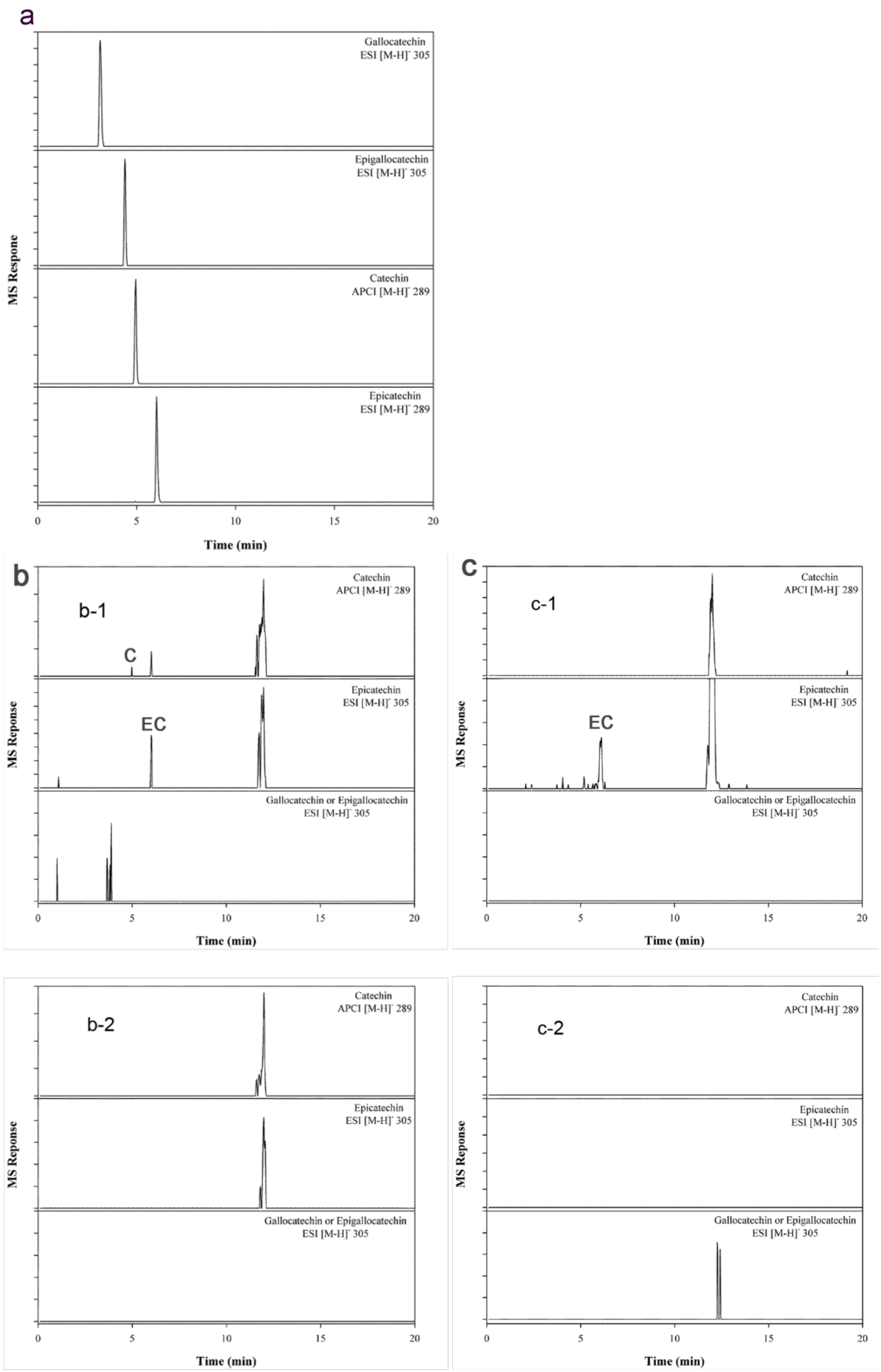

Figure S1. substrate analysis by UPLC-MS. a UPLC-MS profiles show authentic standard of catechin,epcatechin,gallocatecin, epigallocatechin. b UPLC-MS profiles show epicatechin and catechin formed from the incubation of MiANR1-2 and cyanidin(b-1)but not from that of denatured MiANR1-2(control) and cyanidin (b-2), data not shown for MiANR1-3. c UPLC-MS profiles show only epicatechin formed from the incubation of MiANR1-1 and cyanidin(c-1) but not from that of denatured MiANR1-1(control) and cyanidin (c-2)

Kinetic properties of MiANRs

After UHPLC analysis, only catechin and epicatechin were detected from enzyme reaction in which MiANR1-1, 1-2 and 1-3 act as enzymes and cyaniding, perlargonidin as substrates. So standard curve was established for (−)-epicatechin(FigureS2), the standard curve equation for (−)-epicatechin was y = 0.0179x + 0.0339(R^2 = 0.9963). This equation was employed to calculate the production of enzymatic products for optimizing of pH and temperature values and characterization of kinetics.

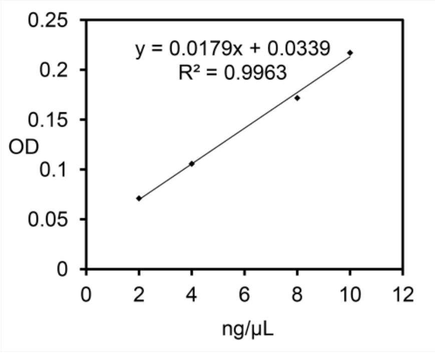

Figure S2. Standard curves of epicatechin. Temperature ranging from 30 °C to 50 °C was tested to get the optima for the enzyme reaction. Results showed all these three enzymes exhibit their utmost efficiency in converting cyanidin to its corresponding flavan-3-ols at 30 °C (Figure S3). Therefore, 30 °C was employed to analyze the kinetic properties .

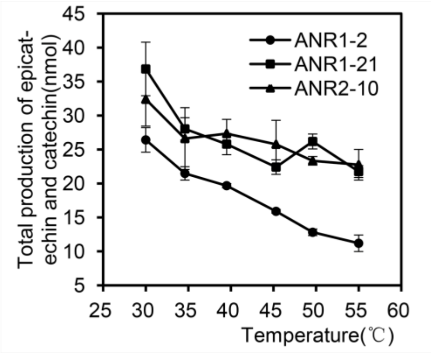

Figure S3. Effect of temperature on MiANR1-1,1-2 and 1-3 activity

Over-expression in the *banuyls* mutant of *Arabidopsis thaliana*

Before transformation, genomic DNA was extracted from leaves of *banylus* mutant 250C and 474C plants and used as templates in PCR to confirm their purity. The primers are as follows: LBb2 5′-ATTTTGCCGATTTCGGAAC.SALK_040250, LP5′-CAATGCCTTCTTTTGTTTTCG, RP 5′-TTCTTCGTTCATCACGATTCC, SALK_122474 LP 5′-TGTTGCAAGCTAAGGAGTTGG.RP 5′-TATTCACAGCCGGAGAATGAG. SALK_040250 LP /RP, LBb2/RP are used to amplify the target DNA in PCR reaction with wild or 250C (mutant) genomic DNA as template respectively. SALK_122474 LP /RP, LBb2/RP are used to amplify the target DNA in PCR reaction with wild or 474C (mutant) genomic DNA as template respectively.

From FigureS4a and FigureS4b, we found by using the three primers (LBb2+LP+RP) for SALK lines, Wild type got a product of about 900bp (from LP to RP), indicating no insertion. 250C lines just obtained one band of about 700 bp in either FigureS4a or FigureS4b, suggesting insertions in both chromosomes. 474C lines got one band f about 700 bp in either FigureS4a or FigureS4b when using LBb2/RP act as primers, however, in FigureS4a 474C lines also got a product of about 900bp when using LP/RP(474C) act as primers, this indicates it is

heterozygous lines.

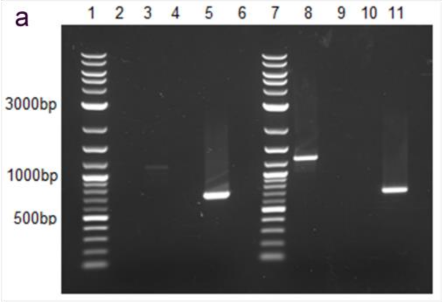

Figure S4a. Identification of *banylus* mutant 250C and 474C plants by PCR

1 DNA ladder. 2 LP/RP(474C) act as primers with wild type genomic DNA as template. 3 LP/RP(474C) act as primers with (474C) genomic DNA as template. 4 LBb2/RP act as primers with wild type genomic DNA as template. 5 LBb2/RP act as primers with (474C)genomic DNA as template. 6 No sample. 7 DNA ladder. 8 LP/RP(250C) act as primers with wild type genomic DNA as template. 9 LP/RP act as primers with (250C) genomic DNA as template. 10 LBb2/RP act as primers with 250C DNA as template. 11 LBb2/RP act as primers with wild type genomic DNA as template.

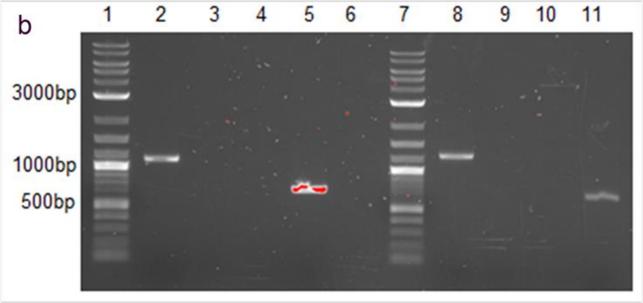

Figure 4S b. Identification of banylus mutant 250C and 474C plants by PCR

1 DNA ladder. 2 LP/RP(474C) act as primers with wild type genomic DNA as template. 3 LP/RP(474C) act as primers with (474C)genomic DNA. 4 LBb2/RP act as primers with wild type genomic DNA as template. 5 LBb2/RP act as primers with (474C) genomic DNA as template. 6 No sample. 7 DNA ladder. 8 LP/RP(250C) act as primers with wild type genomic DNA as template. 9 LP/RP act as primers with (250C) genomic DNA as template. 10 LBb2/RP act as primers with 250C DNA as template. 11 LBb2/RP act as primers with wild type genomic DNA as template.

Molecular docking of MiANRs with NADH/NADPH.

Molecular docking calculations were run using the software Autodock vina and the interactions were generated with PyMol (The PyMOL Molecular Graphics System, Version 2.0 Schrödinger, LLC).

A

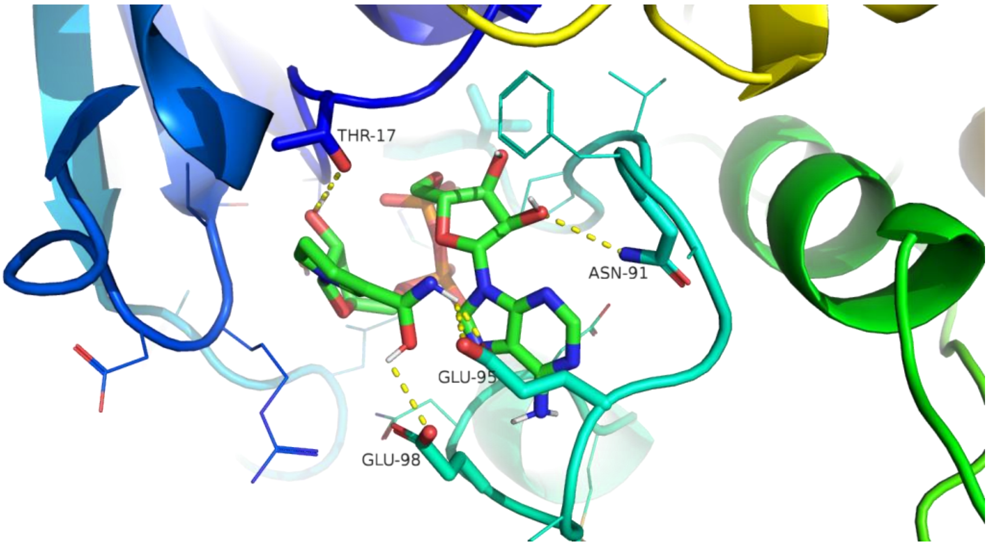
THR-17
ASN-91
GLU-95
GLU-98

B

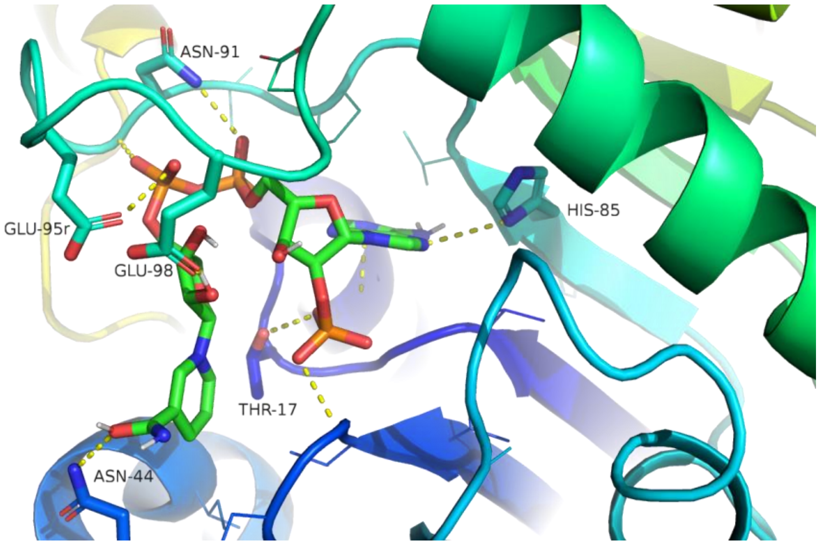
ASN-91
HIS-85
GLU-95r
GLU-98
THR-17
ASN-44

C

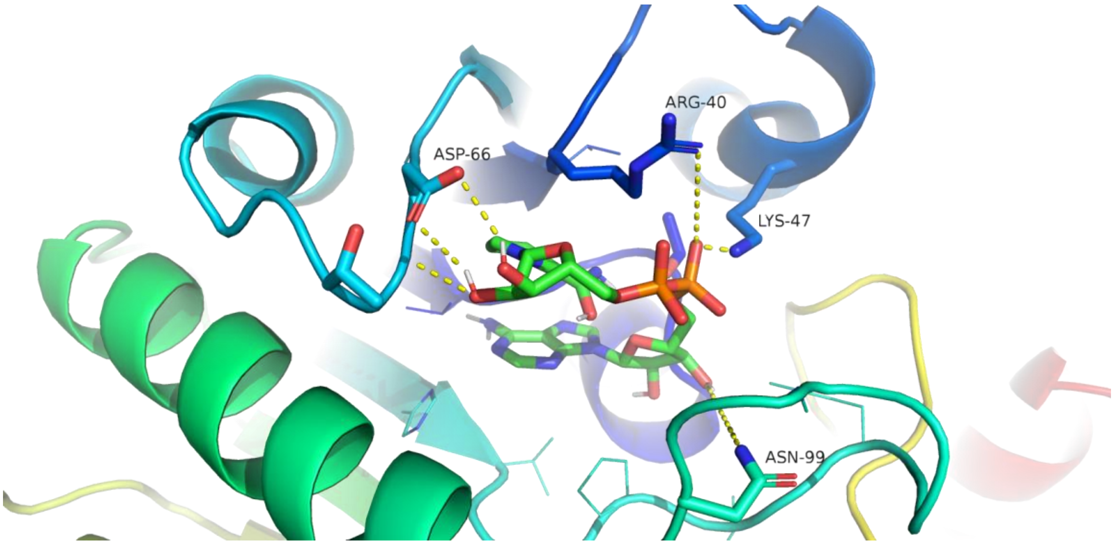
ARG-40
ASP-66
LYS-47
ASN-99

D

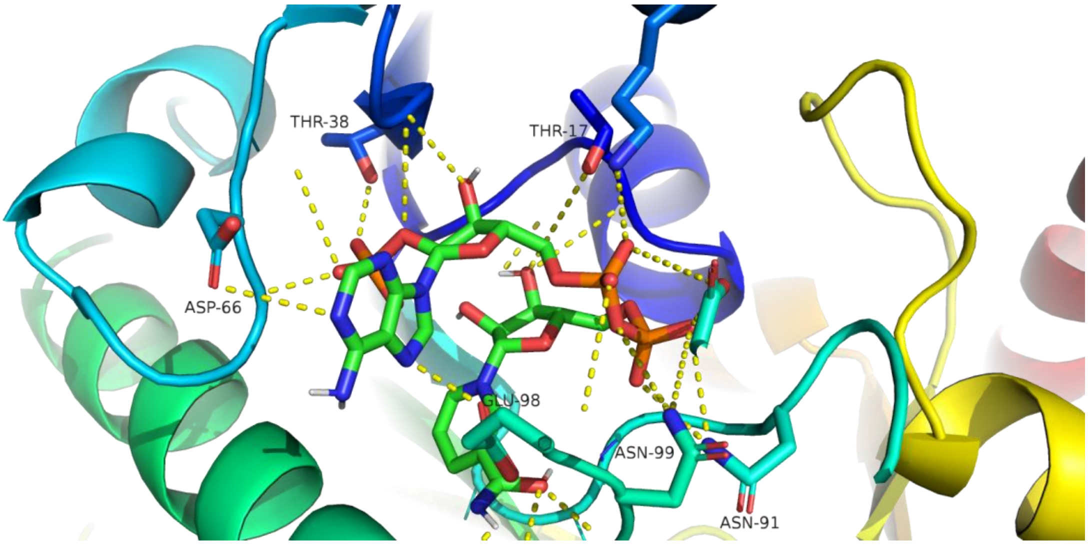
THR-38
THR-17
ASP-66
GLU-98
ASN-99
ASN-91

E

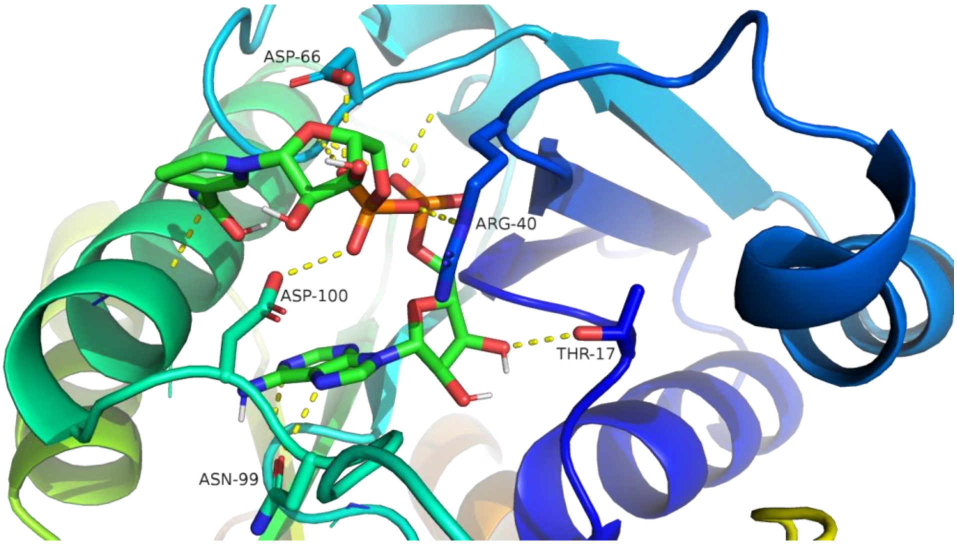
ASP-66
ARG-40
ASP-100
THR-17
ASN-99

F

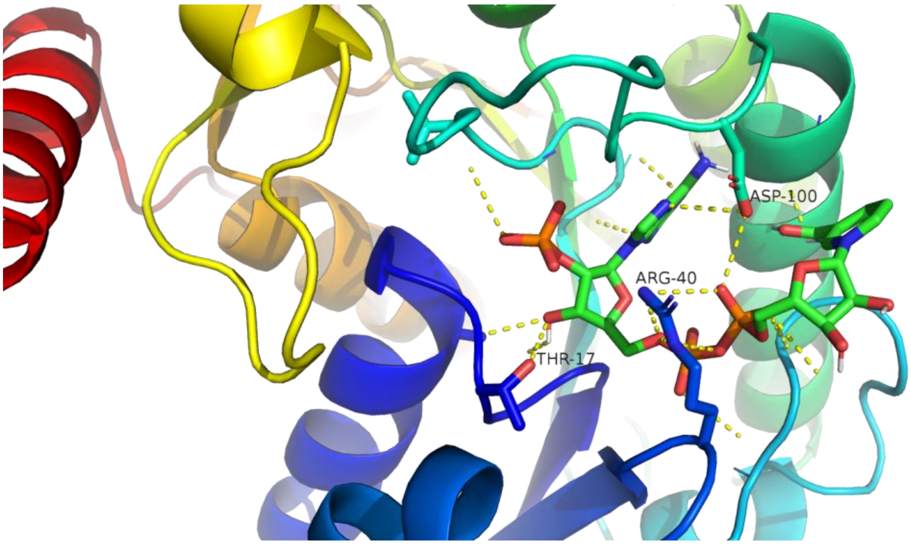

Figure S5. Docking poses for the complex MiANRs with NADH/NADPH. 5SA) Complex MiANR1-1/NADH. 5SB) Complex MiANR1-1/NADPH. 5SC) Complex MiANR1-2/NADH. 5SD) Complex MiANR1-2/NADPH. 5SE) Complex MiANR1-3/NADH. 5SF) Complex MiANR1-3/NADPH. Molecular docking results revealed the formation of a high number of hydrogen bonds between the MiANR1-2 and MiANR1-3 with NADPH, which can contribute to a better molecular interaction. In addition, the binding free energy for the complex MiANR1-2, MiANR1-3 with NADPH was higher respect to the complex with NADH.
